# NanoSolveIT Project: Driving nanoinformatics research to develop innovative and integrated tools for *in silico* nanosafety assessment

**DOI:** 10.1016/j.csbj.2020.02.023

**Published:** 2020-03-07

**Authors:** Antreas Afantitis, Georgia Melagraki, Panagiotis Isigonis, Andreas Tsoumanis, Dimitra Danai Varsou, Eugenia Valsami-Jones, Anastasios Papadiamantis, Laura-Jayne A. Ellis, Haralambos Sarimveis, Philip Doganis, Pantelis Karatzas, Periklis Tsiros, Irene Liampa, Vladimir Lobaskin, Dario Greco, Angela Serra, Pia Anneli Sofia Kinaret, Laura Aliisa Saarimäki, Roland Grafström, Pekka Kohonen, Penny Nymark, Egon Willighagen, Tomasz Puzyn, Anna Rybinska-Fryca, Alexander Lyubartsev, Keld Alstrup Jensen, Jan Gerit Brandenburg, Stephen Lofts, Claus Svendsen, Samuel Harrison, Dieter Maier, Kaido Tamm, Jaak Jänes, Lauri Sikk, Maria Dusinska, Eleonora Longhin, Elise Rundén-Pran, Espen Mariussen, Naouale El Yamani, Wolfgang Unger, Jörg Radnik, Alexander Tropsha, Yoram Cohen, Jerzy Leszczynski, Christine Ogilvie Hendren, Mark Wiesner, David Winkler, Noriyuki Suzuki, Tae Hyun Yoon, Jang-Sik Choi, Natasha Sanabria, Mary Gulumian, Iseult Lynch

**Affiliations:** a Nanoinformatics Department, NovaMechanics Ltd, Nicosia, Cyprus; b School of Geography, Earth and Environmental Sciences, University of Birmingham, B15 2TT Birmingham, UK; c School of Chemical Engineering, National Technical University of Athens, 157 80 Athens, Greece; d School of Physics, University College Dublin, Belfield, Dublin 4, Ireland; e Faculty of Medicine and Health Technology, University of Tampere, FI-33014, Finland; f Misvik Biology OY, Itäinen Pitkäkatu 4, 20520 Turku, Finland; g Karolinska Institute, Institute of Environmental Medicine, Nobels väg 13, 17177 Stockholm, Sweden; h Department of Bioinformatics – BiGCaT, School of Nutrition and Translational Research in Metabolism, Maastricht University, Universiteitssingel 50, 6229 ER Maastricht, the Netherlands; i QSAR Lab Ltd., Aleja Grunwaldzka 190/102, 80-266 Gdansk, Poland; j University of Gdansk, Faculty of Chemistry, Wita Stwosza 63, 80-308 Gdansk, Poland; k Institutionen för material- och miljökemi, Stockholms Universitet, 106 91 Stockholm, Sweden; l The National Research Center for the Work Environment, Lersø Parkallé 105, 2100 Copenhagen, Denmark; m Interdisciplinary Center for Scientific Computing, Heidelberg University, Germany; n Chief Digital Organization, Merck KGaA, Frankfurter Str. 250, 64293 Darmstadt, Germany; o UK Centre for Ecology and Hydrology, Library Ave, Bailrigg, Lancaster LA1 4AP, UK; p UK Centre for Ecology and Hydrology, MacLean Bldg, Benson Ln, Crowmarsh Gifford, Wallingford OX10 8BB, UK; q Biomax Informatics AG, Robert-Koch-Str. 2, 82152 Planegg, Germany; r Department of Chemistry, University of Tartu, Ülikooli 18, 50090 Tartu, Estonia; s NILU-Norwegian Institute for Air Research, Instituttveien 18, 2002 Kjeller, Norway; t Federal Institute for Material Testing and Research (BAM), Unter den Eichen 44-46, 12203 Berlin, Germany; u Eschelman School of Pharmacy, University of North Carolina at Chapel Hill, 100K Beard Hall, CB# 7568, Chapel Hill, NC 27955-7568, USA; v Samueli School Of Engineering, University of California, Los Angeles, 5531 Boelter Hall, Los Angeles, CA 90095, USA; w Interdisciplinary Nanotoxicity Center, Jackson State University, 1400 J. R. Lynch Street, Jackson, MS 39217, USA; x Center for Environmental Implications of Nanotechnologies, Duke University, 121 Hudson Hall, Durham, NC 27708-0287, USA; y La Trobe Institute of Molecular Sciences, La Trobe University, Plenty Rd & Kingsbury Dr, Bundoora, VIC 3086, Australia; z Monash Institute of Pharmaceutical Sciences, Monash University, Parkville 3052, Australia; aa CSIRO Data61, Clayton 3168, Australia; bb School of Pharmacy, University of Nottingham, Nottingham, UK; cc National Institute for Environmental Studies, 16-2 Onogawa, Tsukuba, Ibaraki 305-0053, Japan; dd Department of Chemistry, College of Natural Sciences, Hanyang University, Seoul 04763, Republic of Korea; ee Institute of Next Generation Material Design, Hanyang University, Seoul 04763, Republic of Korea; ff National Health Laboratory Services, 1 Modderfontein Rd, Sandringham, Johannesburg 2192, South Africa; gg Haematology and Molecular Medicine, University of the Witwatersrand, Johannesburg, South Africa

**Keywords:** AI, Artificial Intelligence, AOPs, Adverse Outcome Pathways, API, Application Programming interface, CG, coarse-grained (model), CNTs, carbon nanotubes, FAIR, Findable Accessible Inter-operable and Re-usable, GUI, Graphical Processing Unit, HOMO-LUMO, Highest Occupied Molecular Orbital Lowest Unoccupied Molecular Orbital, IATA, Integrated Approaches to Testing and Assessment, KE, key events, MIE, molecular initiating events, ML, machine learning, MOA, mechanism (mode) of action, MWCNT, multi-walled carbon nanotubes, NMs, nanomaterials, OECD, Organisation for Economic Co-operation and Development, PC, Protein Corona, PBPK, Physiologically Based PharmacoKinetics, PChem, Physicochemical, PTGS, Predictive Toxicogenomics Space, QC, quantum-chemical, QM, quantum-mechanical, QSPR, quantitative structure-property relationship, QSAR, quantitative structure-activity relationship, RA, risk assessment, REST, Representational State Transfer, ROS, reactive oxygen species, SAR, structure-activity relationship, SMILES, Simplified Molecular Input Line Entry System, SOPs, standard operating procedures, Nanoinformatics, Computational toxicology, Hazard assessment, Engineered nanomaterials, (quantitative) Structure–activity relationships, Integrated approach for testing and assessment, Safe-by-design, Machine learning, Read across, Toxicogenomics, Predictive modelling

## Abstract

Nanotechnology has enabled the discovery of a multitude of novel materials exhibiting unique physicochemical (PChem) properties compared to their bulk analogues. These properties have led to a rapidly increasing range of commercial applications; this, however, may come at a cost, if an association to long-term health and environmental risks is discovered or even just perceived. Many nanomaterials (NMs) have not yet had their potential adverse biological effects fully assessed, due to costs and time constraints associated with the experimental assessment, frequently involving animals. Here, the available NM libraries are analyzed for their suitability for integration with novel nanoinformatics approaches and for the development of NM specific Integrated Approaches to Testing and Assessment (IATA) for human and environmental risk assessment, all within the NanoSolveIT cloud-platform. These established and well-characterized NM libraries (e.g. NanoMILE, NanoSolutions, NANoREG, NanoFASE, caLIBRAte, NanoTEST and the Nanomaterial Registry (>2000 NMs)) contain physicochemical characterization data as well as data for several relevant biological endpoints, assessed in part using harmonized Organisation for Economic Co-operation and Development (OECD) methods and test guidelines. Integration of such extensive NM information sources with the latest nanoinformatics methods will allow NanoSolveIT to model the relationships between NM structure (morphology), properties and their adverse effects and to predict the effects of other NMs for which less data is available. The project specifically addresses the needs of regulatory agencies and industry to effectively and rapidly evaluate the exposure, NM hazard and risk from nanomaterials and nano-enabled products, enabling implementation of computational ‘safe-by-design’ approaches to facilitate NM commercialization.

## Highlights

• The variability of nanomaterials makes case-by-case risk assessment impractical.
• Read-across and machine learning approaches are required to ensure consumer safety.
• Access to harmonized and integrated datasets for modelling is a current bottleneck.
• Risk prediction requires integration of models covering release, exposure and hazards.
• NanoSolveIT integrates multi-scale models into an *in silico* risk assessment framework.

## Graphical abstract

**Figure:**
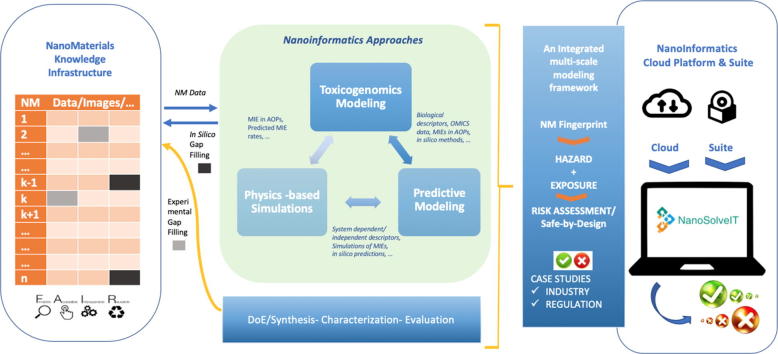

## 1 Introduction

Nanotechnology has increased accessibility to novel, diverse materials with dimensions of ~1–100 nm, the size range within which novel physicochemical (PChem) properties appear and transport processes occur in living systems; such materials are being used in a multitude of industrial and consumer-goods applications. The advantages of engineered nanomaterials (NMs) over similar materials in bulk are well defined; however, in most of the cases, risk assessment (RA) of the potential hazards arising from these new materials properties is incomplete or lacking. Currently, evaluation of possible NM-related risks is an expensive, slow and complicated task, usually achieved by combinations of *in vitro* and *in vivo* experiments that estimate human and environmental hazards. Clear conclusions regarding the hazards posed by NMs are often very difficult because the interpretation of experimental results is influenced by the type of procedures and protocols applied, and studies are usually limited to just a few NMs, doses and timepoints. At this stage, it is clear that proper use of existing high-efficacy occupational technical- and personal protection equipment can be used to sufficiently reduce exposure to NMs, [41], [68], [175] and that the effects from acute exposure to NMs at realistic doses mirror those from anthropogenic particle exposure. However, the challenges now involve assessing long-term low-level chronic exposures, and biological and ecological impacts from multi-component NMs where for example, the different components may degrade at different rates. A validated, predictive *in silico* approach that takes into account the complexity of NMs and the diverse environments in which they are deployed is essential for timely RA and continued progress in nanosafety research.

Despite the great success of computational methods to model and predict properties of conventional chemicals over many years, development of analogous quantitative models relating engineered NMs structure (morphology) to PChem properties and to toxic effects is still underdeveloped, due to:

• The relative paucity of reliable experimental data on the biological properties of NMs;
• Intrinsic complexity of NMs in particle size distribution, shape and degree of agglomeration compared to small organic molecules;
• A limited number of systematic studies on the dynamic interaction of NMs with available macromolecules when placed in a biological environment (such as serum, plasma and environmental compartments) to potentially form coronas which then become the “biologically relevant entity” seen by cells and organisms, and the role thereof.
• Systematic studies conducted to date are limited only to a few nanostructure descriptors and/or physicochemical properties namely, size, shape and surface properties [14], [115].

Despite these limitations, significant progress has been made in recent years towards understanding the drivers of NMs toxicity and ecotoxicity [188], [189]. Size has long been the defining feature of nanoscale materials along with surface area, but is not in itself sufficient as a predictor of toxicity [24]. Surface charge has also been found in many studies to drive toxicity, with cationic NMs being more toxic than negatively charged materials of similar composition, likely a result of strong electrostatic interactions between cationic NMs and negatively charged membranes [42], [77]. Indeed, there are several models linking zeta potential and NMs toxicity in the literature [59], [99], [165]. The presence of crystalline order in materials is another key driver of toxicity, known since the earliest studies with quartz silica and asbestos and applies to NMs such as TiO_2_ and SiO_2_, where amorphous forms have low toxicity while some ordered structures are especially toxic [4], [122]. Band-gap is another common NM property linked with toxicity, as overlap of NM and cellular conductance bands facilitates transfer of electrons and oxidative stress [190]. Surface bond strain arising from high curvature and high temperature synthesis is another feature strongly correlated with toxicity, which explains differences between materials of similar composition produced by different synthesis methods (e.g. with and without high temperatures) [153], [189]. A less investigated parameter that may be important for carbon-based NMs such as CNTs and graphene-like materials is chirality [38]. Many of the properties of NMs are influenced by their surroundings, so-called extrinsic properties, such as dissolution and formation of a biomolecule corona [12], [89]. Binding of specific proteins that facilitate receptor mediated uptake is also a much-investigated feature of NMs, as this drives much of the subsequent signalling [74], [180]. In the environment, transformations such as sulfidation, oxidation and interaction with phosphate, can change NM stability and toxicity [135], [139]. Thus, it is increasingly clear that a wide range of NMs structure descriptors and properties, many of which are interlinked, are correlated with their toxicity. Modelling can elucidate the main drivers of NM toxicity via quantum mechanical and atomistic parameters that provide important insights into reactivity and mechanisms.

Evaluating the hazards of NMs requires the integration and assessment of currently quite disparate NMs characterization and toxicity data, categorization and grouping of NMs, as well as the derivation of exposure routes, forms and concentrations, and hazard threshold levels for human health and the environment. Assessing the hazards of NMs solely based on laboratory tests is time-consuming, resource intensive and constrained by ethical considerations [154]. Consequently, over the past couple of decades, computational approaches for modelling the relationships between NM structure, properties and their biological effects have become a key priority. This field has been reviewed by Winkler et al. [182], but has since advanced further. The most successful computational models, capable of predicting biological properties of NMs in diverse and complex environments, are based on the quantitative structure–activity relationship (QSAR) method. This, and related methods, use statistical and machine learning (ML) algorithms to model relationships between a materials’ structure, molecular properties, provenance and other parameters, and their biological effects. The application of these methods to diverse materials and NMs has been comprehensively reviewed by a number of authors [15], [73], [183], providing some very useful tools. Although data driven, these computational methods are still capable of modelling relatively small data sets [36]. Clearly, larger integrated data sets, and future as yet to be produced data, form the basis for more comprehensive models that will increase automation of experimental data analysis and interpretation. These computational approaches can rapidly fill data gaps, exploit ‘read across’ prediction of biological effects of similar materials, and classify the hazards of NMs to individual species. They are also valuable adjuncts to experimental data on the biological properties of new materials, which remains the bottleneck for reliable risk predictions [1], [55], [97], [172]. Indeed, *in silico* models of this type are used extensively by scientists in academia and industry to reliably calculate PChem properties, and to evaluate effects on human and environmental health, ecotoxicological behaviour and fate of a broad range of chemical substances including complex materials. More importantly, integrating these data resources across disciplines, such as chemoinformatics, systems biology and ‘omics approaches etc., including with non-nanotechnology resources, will support multiple objectives including the reuse of existing information [60]. In addition, this deeper analysis may lead to new discoveries fuelling the innovation pipeline.

## 2 Materials and methods

Below we summarize the latest advances in five important fields of the nanoinformatics sector and how they can be further explored, expanded and incorporated in future generations of NM computational modelling tools and infrastructure. These include: i) dataset curation, quality assessment and knowledge infrastructures, ii) toxicogenomics modelling, iii) multi-scale modelling (physics-based and data driven), iv) predictive modelling (data driven) and v) NMs human and environmental RA. Despite the continuous advances in each sector, researchers face important challenges that need to be solved urgently. These advances are exploited within the European Commission Horizon2020 funded project NanoSolveIT to create the relevant nanoinformatics e-platform to facilitate *in silico* NMs exposure, hazard and risk assessment. To this end, each sub-chapter includes a short analysis and review of the state-of-the-art and recent bibliography, as well as a concise presentation of how the main challenges of each field are being tackled by the authors.

### 2.1 NMs datasets and knowledge infrastructures

Development of nanotoxicity prediction models is becoming increasingly important in risk assessment (RA) of engineered NMs but is dependent on the availability of good datasets with high data quality, completeness [92], and quantity (in terms of numbers of different NMs, but also incorporating a range of doses, timepoints, cell or organism types and end-points). While industry is responsible for the provision of data for their specific NMs for regulatory evaluation, research is required to develop the predictive models to enable reduction of the experimental data needed for regulatory RA. However, computational researchers face significant challenges in acquiring this data needed for the development of robust models. These have included the wide heterogeneity of published literature data in terms of NMs characterization, exposure and hazard data reported, the availability of the underpinning datasets in a form useful for modelling, and in regards to the variable degrees of completeness and quality of available datasets [92], [157]. Therefore, in the field of nanoinformatics, there has been special emphasis on the curation and quality assessment of nanosafety data. A small number of recent projects have thus prioritized the curation of literature data and datasets generated in past projects that ended before the current (evolving) standards for data quality emerged (e.g. NanoReg2 and caLIBRAte projects curating NaNoReg data), NanoSolveIT partners curating literature publications on NMs transcriptomics etc.).

Diversity of experimental approaches in the literature data has led to many missing values (data gaps) and differing data quality (numbers of replicates, signal to noise ratio, relevance of endpoints, different experimental conditions etc.), such that assessment of the quality and completeness of the collected data is a critical issue for modellers and regulators [92]. Previously, Klimisch et al*.* [65] proposed criteria to assess the reliability of toxicological and eco-toxicological data based on the source of the toxicity data (i.e. whether the data were produced using international standard operating procedures (SOPs) such as the aforementioned OECD test guidelines). Thereafter, Lubinski et al. [83] expanded on these criteria to include PChem properties of NMs such as size, shape, and surface charge. Recently, Ha et al. [45] and Trinh et al*.* [168] further extended criteria for evaluation of the quality and completeness of NM’s PChem data. The quality and completeness of these data were determined using a set of rules which specifically assigned a PChem score for each attribute reported (i.e. core size, hydrodynamic size, surface charge and specific surface area). The score for each attribute was composed of two sub-scores; one for the reliability of the data source (score range: 0–3) and another for the reliability of the measurement method (score range: 0–2). To evaluate the quality and completeness of a dataset, the average values and standard deviation of scores of all data rows in a dataset are used [168].

#### 2.1.1 Data generation and curation activities

To support the development of better prediction models, there has been several data generation and data curation and quality assessment activities reported. For instance, Hendren et al. [50] introduced the NMs Data Curation Initiative almost 10 years ago, and explored the critical aspect of data curation within the development of informatics approaches to understand the behaviour of NMs, while Powers et al. [130] proposed a workflow for nanosafety data curation and Robinson et al. [92] discussed various issues in the evaluation of curated NM data, such as considering its completeness and quality, where the requirements for each will depend on the purpose for which the data was generated and/or will be re-used. Examples of experimentally generated and literature mined datasets suitable for modelling have also recently emerged. For example, Puzyn et al*.* [131] performed an experiment to determine toxicity of 17 different types of metal oxide NMs towards *E. coli* and proposed a simple equation for prediction of the effective concentration at which 50% of the organisms were killed (EC_50_) using the enthalpy of formation of a gaseous cation. Walkey et al. [177] published experimental data and a model utilizing protein corona fingerprints to predict NMs cellular attachment, consisting of 105 surface modified gold NMs, although it turns out that the corona isolation method utilized here inadvertently removed albumin which is usually a major constituent of NM protein coronas [76], [177]. Furthermore, Oh et al. [118] conducted a meta-analysis of more than 300 published articles reporting the toxicity of quantum dots and found that only a few parameters - surface properties, diameter, assay type, and exposure time, contributed significantly to their toxicity. Gernand et al. [40] collected and analyzed literature data on the rodent pulmonary toxicity of uncoated, unfunctionalized carbon nanotubes (CNTs) and proposed that the main factors driving pulmonary toxicity of CNTs were metallic impurities, CNT length, CNT diameter, and aggregate size. Melagraki *et. al.* [97] used *in silico* methods to investigate published datasets, constructing and validating a predictive model using an organized dataset on NMs cellular uptake of 109 NPs tested in pancreatic cancer cells (PaCa2). Recently, Ha et al. [45] extracted and compiled a dataset for 26 metal oxide NMs from 216 literature articles related to the toxicity of metal oxide NMs. Trinh et al. [168] on the other hand, collected cytotoxicity data of metallic NMs, which includes PChem properties, their measurement methods (PChem attributes), *in vitro* cytotoxicity assay conditions and resultant cell viability data (Tox attributes). Each of these datasets has been, and continues to be, re-used in the development of predictive models for NM toxicity prediction.

Importantly, a number of large EC and internationally funded projects were recently completed (e.g. [29]), describing large libraries of well characterized NMs and their accompanying hazard and/or exposure datasets. Table 1 lists these datasets, which are in various stages of curation and ontological annotation for semantic mapping and database integration, by project: eNanoMapper, NanoMILE, NanoSolutions, NANoREG, NanoReg2, caLIBRAte, NanoTEST, NanoFASE, the Nanomaterials Registry, the 2 NSF-funded centers for environmental implications of NMs (CEINT and CEIN) and South Korea’s S2Nano, for which several relevant PChem and biological endpoints have been assessed mostly using OECD methods.

**Table 1:** Datasets from various sources contributed by NanoSolveIT partners which are currently being curated and ontologically annotated by NanoSolveIT for use in modelling and federation into a knowledge commons.

Projects	Materials	Information included	Numbers
NanoMILE	Diverse NMs (i.e. ZnO, CuO, Au, Ag, CoO, SiO_2_, BaTiO_3_, AlOOH, Si, CeO_2_, CuO, hydroxyapatite) with nanoinformatics and *in vitro* toxicity data	Size dependent nanodescriptors	>3000 datapoints
NanoSolutions	A panel of 30 industrial NMs each with variants of surface – uncoated, positive, negative and PEG coated. Also CNTs, Nanocellulose and more.	Omics and intrinsic properties on NM *in vitro / in vivo* effects *in vitro* high throughput screening	>100 NMs 31
SmartNanoTox	TiO_2_, SiO_2_, Au, carbon nanotubes etc. (binding free energies and potentials of mean force for interactions for all)	Interactions of amino acids and components of lipids and sugars with NMs (computational and experimental data)	>5 NMs
NanoFASE	TiO_2_, CeO_2_, Ag, Ag_2_S	Transformations of NMs in the environment (air, water, sediment, soil, waste treatment and biota) and release models	
Nanomaterial Registry	Diverse NMs	NanoMaterials Registry Database	>2000 datapoints
NanoTEST	TiO_2_, two fluorescent SiO_2_, Iron oxide coated and uncoated, PLGA	Genotoxicity, cytotoxicity, uptake, oxidative stress	>5000 datapoints
S2NANO	Various engineered NMs (e.g., Oxide NM, Metallic NMs, and Carbonaceous NMs) 28–30 Curated from literature and experimental studies.	PChem properties / characterization; cytotoxicity assay conditions.	16 NMs datasets
CEINT NIKC	Ag/Ag_2_S, CuO, Graphene oxide, CNTs, CeO_2_, nZVI, cellulose nanocrystals, TiO_2_, gold etc. – literature curated datasets / mesocosm datasets.	NM intrinsic, extrinsic (system dependent), social (e.g. use scenarios, matrix, concentration in products) properties; System characteristics; Exposure / Hazard data; Meta-data (protocol, temporal and spatial descriptors etc.)	20 NMs datasets
UC-CEIN NanoDatabank	Pristine MOx NM, quantum dots, CNTs/graphene 300 toxicological assessments, 150 investigations (curated data from over 500 publications)	PChem properties / characterization; toxicological assessments; NM fate, transport and material characterization.	->1000 NMs
Modern	metal oxide NPs of 12 sizes between 5 and 60 nm	35 full particle nano-descriptors	24 NMs 10,080 datapoint
NanoTOES	Ag NMs: 3 different sizes same surface properties, Ag NMs 20 nm with 6 different surface properties	PChem properties / characterization; cytotoxicity and genotoxicity endpoints;	9 NMs, >1000 datapoints
eNanoMapper	Publicly available datasets included	PChem properties / characterization; Hazard data	636 NMs, ~1750 datapoints

Based predominantly on OECD documents from 2005 to 2017, Steinhauser and Sayre reviewed and summarized the key PChem properties, their preferred measurement metrics, as well as strengths/weaknesses of intrinsic and extrinsic properties (where they go, i.e. persistence, or, what they do, i.e. reactivity) in terms of predicting NMs behaviour [151]. The major intrinsic properties that they focused on included particle size distribution (number average), particle shape (e.g. aspect ratio), surface area, redox potential/band gap, crystalline phase(s), hydrophobicity, chemical composition (impurities, surface chemistry), and, rigidity. The extrinsic properties related to persistence included biodurability, zeta potential, density, dustiness (depends on moisture), dissolution rate (in environment, acellular), agglomeration/hydrodynamic diameter (dispersion stability) and surface affinity. Those related to reactivity only included reactive oxygen species (ROS) production and photoreactivity. A key factor in determining the extrinsic properties is the need to characterize the NMs in the relevant exposure medium and across the relevant exposure times [87], [89].

#### 2.1.2 Computational approaches for data gap filling and interpolation

Technological advances including high content and high throughput screening and omics approaches have transformed nanosafety research into a data rich field. Concurrently, nanoinformatics and ML-based *in silico* modelling applied to nanosafety require even larger data sets. For NM property models to be robust, predictive, and broadly applicable, large amounts of high-quality and complete experimental data are needed, that are organized and accessible. A current bottleneck is the fragmentation and inaccessibility of much of the data generated to date. To overcome this data fragmentation and facilitate model development, new processes need to be developed and implemented that will allow the capturing of both the NM data and the associated metadata making the produced datasets findable, accessible, interoperable and re-usable (FAIR) [54], [181]. This two-fold process aims at extending existing nanosafety and nanoinformatics databases with interfaces that allow curation, ontological annotation and semantic mapping of the data schema, and extraction of data and knowledge about NMs, as well as implementation of very focused set of experiments, designed to fill gaps identified in the existing large datasets. The combination of these activities will support the development of computational predictive toxicology methods, enable *in silico* models to perform at optimum levels, and increase the predictive power of the overall Integrated Approaches to Testing and Assessment (IATA) for human and environmental RA that is envisioned within NanoSolveIT. A strong focus is given to the production of curated, reliable NMs safety data under the FAIR principles [181].

Among the approaches available to overcome data fragmentation and data inaccessibility is data mining from literature and subsequent meta-analysis of the curated data [5], [71], although this can be extremely time consuming if done manually. Text mining algorithms are under development [44], [78] and their suitability for NMs is being evaluated within other ongoing nanosafety data projects such as NanoCommons. One issue arising from these literature mining approaches is the degree of data quality and completeness [83], [92], as well as the comparability of datasets generated by different groups using different batches of NMs [102] or characterization protocols. More specifically, data quality concerns originate from the different methods/protocols and experimental conditions used for the data production (e.g. lack of characterization in the exposure media) and the lack of sufficient metadata to describe the data and ensure interoperability. Data completeness concerns are linked with the amount of PChem characterization of NMs performed prior to and during the experimental procedure in the relevant exposure medium, and the respective endpoints (toxicological, omics etc.) measured. The impact of these factors on modelling can be increased data variance and unreliability and decreased model robustness and predictivity. This is especially true in the development of nano-QSAR/nano-QSPR (quantitative structure–property relationship) models, where the variability observed in the PChem properties of NM might be higher than it is in reality [83].

To overcome these issues several approaches have been proposed [36], [37], [45], [90], [168]. For example, Ha et al. [45], demonstrated the benefits of data gap filling during the meta-analysis of the cytotoxicity of metal oxide NM using data mined from 216 publications, which resulted in 6,842 data rows and 14 attributes of nanostructure descriptors, PChem, toxicological and quantum–mechanical (QM) (computational) properties. Gap-filling was achieved using information from manufacturers’ specifications, references utilizing the same NM or with estimation from other PChem properties. Data quality was assessed using a scoring system based on the presence and origin of data. Gajewicz et al*.* [36], on the other hand, proposed the use of read-across algorithms to predict the missing values and improve the predictive outcome of predictive models. In all cases, data gap-filling and increased data quality resulted in increased accuracy of the nano-structure–activity relationships models.

For such methods to be successful, detailed workflows for the experimental and literature data curation are needed [130]. Similarly, standardized experimental workflows, and complete reporting of experimental procedures including computational and database mining, need to be established to ensure data reproducibility at a wider scale [17], [76], [137]. NanoSolveIT aims to develop the gap-filling approaches and curation workflows through the gathering of existing datasets originating from recently completed EU funded projects (e.g. NanoMILE, NanoFASE, caLIBRAte, NanoTEST and others reported in Table 1 above), performing the necessary evaluation of the existing data and metadata and designing detailed experiments to fill the identified gaps in the NM PChem characterization and toxicity endpoints, thus increasing their quality. The resulting larger datasets will then be used by the modelling partners for the development of more robust *in silico* approaches with wider domains of applicability and enhanced predictive capability, while the developed standardized workflows will be made available for experimentalists to guide them in the production of the necessary scale and completeness of data for use in modelling approaches.

#### 2.1.3 Dedicated NMs databases organized for modelling and informatics

The NanoSolveIT knowledge base will extend the NanoCommons / eNanoMapper databases with innovative, ontology-based application programming interfaces (APIs) to allow semi-automated curation and extraction of data and knowledge about NMs to support development of computational predictive toxicology methods. It will cover a wide range of data that researchers are looking for: omics data, nanodescriptors and relevant literature, as well as PChem properties and biological effects. There are two key aspects of the knowledge base. Firstly, NMs-specific datasets will be federated with other databases that are optimized for endpoints from proteomics, transcriptomics etc. for which well-established data management and deposition solutions already exist. The NanoSolveIT knowledge base will communicate via APIs and integrate the data via semantic mapping of their data schemas. Secondly, the knowledge base will be enriched by integration of data from protein structures, known signalling pathways, crystal structure information, for example. These data will be used by physics-based modelling approaches (see Section 2.3) to computationally design NMs and their biomolecule fingerprints. Other types of data that can be enriched in the knowledge base include environmental data such as river pH, ionic strength, dissolved organic matter etc. which can support development of enhanced models for prediction of NM’s environmental transformation and consequent ecotoxicity.

Furthermore, the NanoSolveIT knowledge base will adopt Open Science approaches to trigger open innovation with related projects and future users and collaborators. This will result in the NanoSolveIT Knowledge Infrastructure containing curated, reliable NMs safety data, accessible and reusable within the project, by the nanoinformatics community and by all stakeholders. The NanoSolveIT knowledge base will be helpful for those researchers interested, not just in the structural characteristics (descriptors) and PChem properties, but also in the known biological effects of particular NMs. Researchers can use the interface to navigate through the available data or retrieve data for further exploitation.

### 2.2 Toxicogenomics modelling (predictive models using omics data)

Toxicogenomics modelling is a subdiscipline of pharmacology that deals with information about gene and protein activity within a particular cell or tissue of an organism in response to exposure to toxic substances. It can be used to link the safety of NMs to the underlying biological mechanisms of their toxicity. Gene expression data can be combined with biological pathway information to identify possible adverse outcomes [43], [67], [110]. In order to make sense of the large volume of data generated by bioinformatics analyses, SOPs for the interpretation of the results in the correct biological context need to be established [46]. Adverse Outcome Pathways (AOPs), which describe in mechanistic detail the sequences of events that are necessary for an exposure at cellular and subcellular levels to lead to an adverse event or outcome at the organ or organism level, are a useful tool for organizing bioinformatics and other types of results into a predictive framework [111].

During the last decade, multiple efforts have aimed at characterizing the mechanism (mode) of action (MOA) of toxic chemical exposures using transcriptomics profiling of the exposed biological systems. This generated large reference data sets such as connectivity map [152], TG-GATEs [51], DrugBank [184] and LINCS L1000 [66]. These have been extensively used for drug repositioning (e.g. [53], [106]) and toxicity prediction (e.g. [67]). The general concept of this approach is that toxicogenomic experiments identify the primary molecular changes in cells and tissues as a direct consequence of toxin exposure, and hence directly inform the toxicity pathways of tested compounds [43]. As an example, Predictive Toxicogenomics Space (PTGS) components, which were developed based on connectivity map data to predict organ toxicity, are likely useful as descriptors of AOP-linked MOAs and key events (KEs) in the affected signalling pathways [67].

Furthermore, when multiple time points are screened, toxicogenomics data can give a robust insight into the toxicokinetics and can further assist the drafting of an AOP [35], [111], [185]. Toxicokinetics describes the absorption, distribution, metabolism and storage/excretion of chemical toxicants, while, toxicodynamics describes the adverse (biological) effects that a toxicant has on an organism, e.g. altered structure/function and disease. Both processes are determined by the structure (morphology) and PChem properties of NMs, e.g. size, shape and surface reactivity [95]. Toxicogenomics data can also be exploited to infer similarities between different types of exposure and between exposure and human diseases [145]. Finally, new opportunities are emerging for integrating ‘omics data with intrinsic NMs exposure properties to allow hybrid quantitative structure and MOA activity relationships to be developed [146].

More recently, toxicogenomics approaches have been employed to describe the MOA of NMs in various exposure scenarios *in vitro* (e.g. [63], [101], [144]) and *in vivo* (e.g. [47], [52], [64], [70], [107], [132], [141]). When multiple doses of toxin are screened by ‘omics technologies, dose-dependent events can be extrapolated in order to further dissect specific mechanisms of toxicity [133], [158]. In fact, dose metrics are a basic requirement for any *in vitro* screening to assess potential health risks of NMs. Genomic dose responses can be used to define the biological potency of a material as well as points-of-departure concentrations denoting adverse levels of exposure to the organism or cell. Typically point-of-departure concentrations are set using concentration response modelling or the lowest observable effect level approach [158]. Although transcriptomic response itself is often triggered as a response to an adverse reaction to the environment, a pathway-level concentration is typically used, as this value is more robust than a gene-level response and is directly connected to a known biological response. Further modelling methods, such as the PTGS, can be helpful to differentiate between adverse, adaptive and benign (or even beneficial) biological responses to chemicals. Mechanisms or pathways connected to key event responses in known AOPs are also applied for selecting adverse responses among bioinformatics analysis results [111]. When cell culture data is utilized, there is also the need to extrapolate the cell culture concentration to a biological exposure scenario which in the case of NMs is typically inhalation-based [111]. However, focusing on mechanistic aspects, the pathway-level benchmark concentrations can be used to rank NMs based on biological potency. Taking the concentration response into account is also helpful for biological grouping, e.g., for selecting optimal treatments for connectivity mapping-based biological similarity analyses. As the cost of toxicogenomics is steadily being reduced, its use in safety assessment and mechanistic analyses will only grow in importance.

**Fig. 1:**
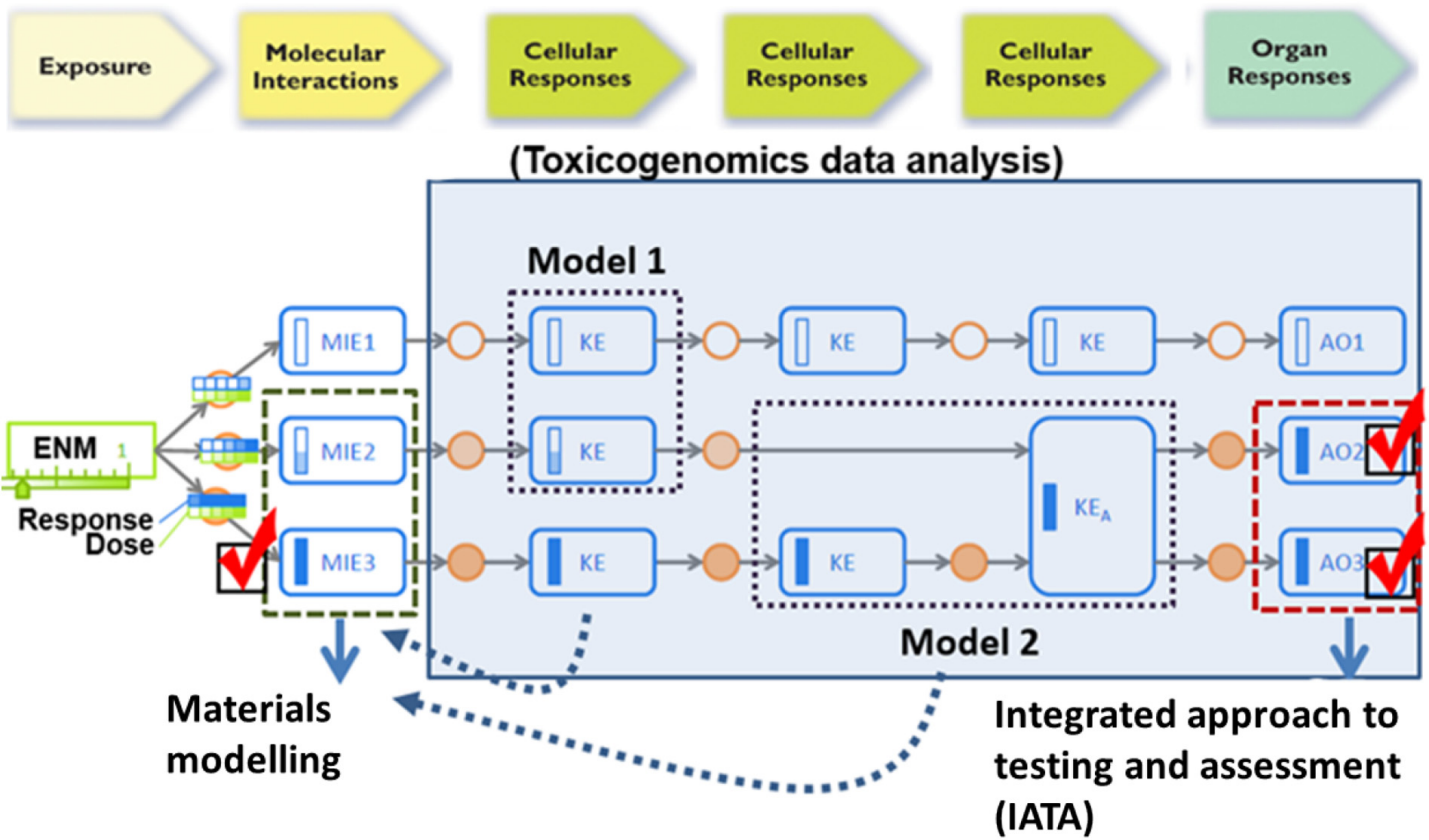
Schematic overview of the workflow for toxicogenomics modelling and how these models feed into the subsequent materials modelling and IATA. AO – Adverse Outcome; ENM – Engineered Nanomaterial; KE – Key Event; MIE – Molecular Initiating Event.

NanoSolveIT is collecting existing toxicogenomics data, then transforming, analyzing and modelling it. The project has multiple aims, including deriving signatures of NM MOAs to support AOP development; generating useful predictive models of the biological effects of NMs; and developing computational methods and software to be included into a robust computational platform for *in silico* nanosafety analysis (as seen in Fig. 1).

#### 2.2.1 Deriving signatures of NM MOA to support AOP development

Toxicogenomics has provided unprecedented opportunities to clarify the MOA of many chemical exposures. The essential idea is to identify a set of genes that are significantly altered in a given biological system of interest due to exposure to a toxic agent. While conventionally, ‘omics data analysis resulted in lists of differentially expressed genes, these are not *per se* informative of more complex patterns of regulation that underlie broader biological functions. To elucidate these functions, systems biology approaches, such as gene network reconstruction and inference, have been used to identify complex patterns of molecular regulation and co-regulation [64], [192]. Moreover, the systematic annotation of molecular changes into known biological pathways has helped define toxicity and other AOPs [70], [111], [144]. To date, the transcriptome has been the primary molecular focus of such studies, followed by proteomics and metabolomics. Multi-omics approaches have already been used extensively to generate more thorough landscapes of molecular changes in human diseases (e.g. The Cancer Genome Atlas) and are beginning to be used to build more general models of NM MOA [144]; [143]. Omics-derived biosignatures are valuable for comparing the effects of different types of exposure, such as NMs and small molecules that would otherwise be difficult or impossible to detect by other means. For example, omics-derived NM biosignatures have been systematically compared to those from small molecules, drugs and human diseases in search of direct exposure-disease associations [145]. These analyses have identified biomarkers useful for biochemical assays in the zebrafish model, enabling the drafting of an AOP for metal and metal oxide NMs impacts on the central nervous system [75].

#### 2.2.2 Deriving signatures of NM MOA that define robust predictive models based on NM bioactivity

Toxicogenomics data has more recently been used to generate predictive models of toxicological and pharmacological interest. Multiple strategies have been employed: identification of specific biomarkers [145], or discovery of broader gene sets with strong predictive ability (PTGS, zebrafish-based toxicogenomic space, etc.). Typically, ‘omics data analyses use univariate statistical testing, where each molecular feature is tested for significant differences between exposed and unexposed sets in replicate samples. However, these approaches allow derivation of only very limited individual or linear or concatenated biomarkers. The output from these analyses, importantly, does not guarantee biomarker specificity, although they are often referred to as such in the literature. Thus, more sophisticated feature selection strategies are needed, that allow non-linear combinations of molecules, or sets of biomarkers that are more specific. To this end, a number of algorithms have been proposed including GALGO [138] DIABLO [149], MANGA [145], MLREM [138], [167], [9], [34], [6], [147].

#### 2.2.3 Developing computational methods for inclusion into a robust computational platform

Despite the great value of toxicogenomics approaches in identifying important (NM) toxicity mechanisms in an unbiased manner, these approaches have thus far struggled to be implemented in the mainstream regulatory framework for chemical safety assessment. One reason for this is that toxicogenomics data are usually difficult to interpret without strong skills in bioinformatics and biostatistics. Importantly, the scientific community has been successful in developing improved analytical methods but has not yet agreed on formalized standard analytical SOPs. Clearly, omics data analysis is used extensively in other biomedical research fields, so many of these standard methods should be transferrable to NM toxicogenomics problems. The NM toxicogenomics community needs to ensure that this existing expertise is converted into standardized pipelines and software for nanosafety applications. Examples of useful methods are the eUTOPIA software for omics data preprocessing [94] and INfORM for gene network inference [93]. Similar efforts are being undertaken, within NanoSolveIT and several other EU projects, to further resolve dose dependent patterns of molecular change and to benchmark the resulting toxicogenomics and AOP models to increase their utility and acceptance by the community.

### 2.3 Multi-scale modelling framework for NMs property prediction

Adverse human health effects can be triggered and modulated by molecular-level interactions at the bionano interface, i.e. a nanoscale layer where biological entities meet foreign materials. These interactions are often non-specific and unintended. The currently poor understanding of the bionano interface means that RA for NMs and biomaterials broadly is largely based on empirical evidence and not on the mechanistic action of the adverse effects. In general, NM properties primary responsible for adverse effects are largely unknown, or are not the same as the PChem properties that can be routinely measured [89], [136]. Understanding these interactions and the bionano-interface structure will assist with developing safety regulations and reducing the associated health risks but also with achieving improved control over the surface activity in nanotech-based applications.

Steinhauser and Sayre [151], reviewed the OECD guidance documents for NMs risk assessment, which provided recommendations regarding the measurement and assessment of occupational exposure, consumer exposure, environmental fate, ecological effects and biokinetics, as well as considerations for *in vitro* testing of NMs, for increased reliability and relevance. However, of particular interest for NanoSolveIT were the guidance documents for exposure modelling and QSAR modelling. While QSAR models usually employ two-dimensional (2D) structural information from molecules, they can employ three-dimensional (3D) information also, making them suitable for predictions of NMs properties and behaviour. The benefit of 2D is that it provides a good visualization of the structure, where one can easily identify the connectivity of atoms, the presence of specific functional groups and predict reactivity. However, 3D focuses on the molecular level and includes additional information related to bond distances or angles, as well as connectivity or binding to ligands in relation to surface topography and can account for extrinsic properties such as formation of a protein (biomolecule) corona, for example.

#### 2.3.1 Computational NMs descriptors

In addition to direct correlations between the NM structure (expressed in term of nanostructure descriptors), properties and toxicity, as described above, interactions at the bionano interface can initiate AOPs via sequestering or unfolding of proteins central to molecular initiating events (MIEs) and KEs of the corresponding pathways [21], [32], [86], [88]. Although they may not be completely independent of the basic features of the NM (as expressed either directly by nanostructure descriptors or by their intrinsic properties), a systematic evaluation of these types of properties that express protein affinity, protein unfolding and potential formation of cryptic epitopes that can induce new signalling pathways [86], may make predictive models more compact and robust.

Simply stated, a *descriptor* is the final result of a logic/mathematical process that transforms chemical-based information (encoded within a symbolic representation of a chemical structure) and, in the case of NMs, also physical-based information (i.e. morphology) into a useful number that can be exploited by a predictive model. Thus, descriptors provide unique information required to draw (or build a molecular model of) a NM. Whereas *property* (e.g. solubility) should be considered as a consequence of the NM’s structure; it is impossible to deduce back the structure from such properties only. The properties can be either measured experimentally or computed with use of first-principles-based methods (e.g. *ab initio*, Density Functional Theory, molecular dynamics). The correct distinction between *descriptors* and *properties* is important, since only the structure (descriptors) can be directly controlled by a designer in the safe-by-design process.

There are various levels for chemical structure representations (descriptors) ranging from one-dimensional (1D) descriptors (e.g. basic molecular formulas), to 2D descriptors (e.g. connectivity index), as well as 3D that are conformation dependent (e.g. dihedral angles, radius, shape).

Examples of computational properties based on NMs interactions that can be related to MIE, KE or AOPs include: composition of the NM protein corona; adsorption enthalpy of amino acids, lipid molecules, or proteins onto the NM surface; adsorbed protein or NM hydrophobicity; production of ROS, dissolution of NMs leading to release of ions, all of which must be determined in realistic environments [20]. Calculation of properties based on a full-particle molecular model, using *ab initio* quantum chemical (QC) or even semi-empirical methods remains unfeasible in the near future due to the large size of NMs and thus the associated enormous computational time needed. Therefore, a significant effort was invested by the community to develop different approximations and simplified molecular models of NMs to derive NMs properties.

One of the first approaches for calculation of nanodescriptors was the design of optimal molecular descriptors by Toropov et al. [162], [164], [166]. These descriptors are calculated from SMILES structures and consider the chemical composition of NMs. Information about experimental conditions of NMs synthesis can be included in the descriptor calculation. This type of descriptor has been successfully used to model the toxicity of NMs [162], [166].

QC calculations based on small clusters of atoms can be used to obtain such properties as HOMO-LUMO gap (band-gap between conductance and valence electrons) and enthalpy of formation, which is currently not possible for full-sized NMs. QC properties can be directly used to model toxicities of NMs without taking into account the size dependency of the properties [131] or can be extrapolated to obtain properties values for specific size of NMs [56]. To take into account the effect of size on the properties of NMs, the calculations based on so-called full-particle molecular model should be performed. Such type of calculations for metal oxide NMs have been performed by [156], [155], [91], [11]. Full-particle properties were derived from molecular mechanics calculation of NMs and describe the energetics (potential energies), coordination numbers and other attributes of the NMs. Full-particle descriptors and properties have been successfully used to model the toxicity of metal oxide NMs [156].

Surface modifications, such as change in coating materials, influence the properties of NMs and should also be included in the modelling process. Xia et al*.* [186]) proposed a biological surface adsorption index to describe competitive adsorption of proteins onto the surface of NMs [187]. The adsorption coefficient is expressed as a logarithmic function of five descriptors: excess molar refraction (representing molecular force of lone-pair electrons); the polarity/polarizability parameter; hydrogen-bond acidity and basicity; and the McGowan characteristic volume describing hydrophobic interactions [186]. Experimentally obtained log K values can be used to derive five descriptors for surface forces related to adsorption. Combining all of these approaches would allow the characterization and modelling of NMs in biological systems accounting for all important aspects (electronic effects, size dependency and surface modifications) and would greatly improve the quality of nanoQSAR models.

#### 2.3.2 Modelling (predicting) NM corona composition

The structure of the bionano interface can be simulated using first principles physics-based methods. Such simulations are often computationally intractable or use model systems that do not capture the complexity of real biological environments [72]. The relevant system sizes are too large for direct atomistic simulation, so the properties of interest can only be accessed using a coarse-grained (CG) representation, where sub-nanometer interactions have been integrated out. Molecular details of the NM are preserved when the CG model is constructed using a multistep approach, where each layer is parameterized from simulations at finer resolution [129]. Atomistic simulations also have challenges as they rely on accurate and validated force fields. Where such force fields are available, all-atom Molecular Dynamics (MD) methods can be used to construct a united atom representation of the NM and associated biomolecules. Computational study of new materials requires development or optimization of new atomistic force fields based on QC calculations or experimental data [8].

Coarser scale simulations also have challenges due to the nature of biological samples: the number of relevant biomolecules interacting with the NM can be enormous; for example, human plasma contains over 3,700 proteins and even larger numbers of metabolites [16]. The corona composition (lists of proteins and metabolites (lipids and other small molecules) known to interact with a specific NM) may therefore be an impractical property to be used for predictions, although meta-analysis of over 63 NMs plasma corona studies suggested that about 125 proteins form the interactome of NMs [176]. Each NM immersed in plasma typically has its own unique corona that may involve hundreds of different proteins [13], [23]. Proteins in the corona reflect the functionalities on the NM that bind specific types of biomolecule [85]. This changes over time, as the most abundant proteins bind first, and are subsequently replaced by less abundant but more tightly bound proteins, and the corona also evolves as the NMs are internalized into cells for example [84], and the cells respond to the presence of the NMs [2]. Capturing this complexity in descriptors used in ML models is very challenging, and often statistical properties of descriptors for the proteins are used.

Early examples of corona-based predictive schemes exist in the literature. An extensive gold NMs protein corona dataset generated and analyzed by Walkey et al*.* [177], was re-analyzed in order to identify and quantify the relationships between NM-cell association and protein corona fingerprints in addition to NM PChem properties [1], [80]. QSAR models were developed based on both linear and non-linear support vector regression models making use of a sequential forward selection of predictors. For example, an initial pool of 148 predictors was used, with the analyses eventually identifying 10 corona proteins and 3 PChem characteristics (NM size and zeta potential in cell culture medium) as the most significant factors correlating with NM cell association [1]. As more data emerges, including on the small molecule or metabolite corona and how these interact with proteins to form the complete corona [16], refined models and more detailed predictions of the composition and impact of the NM corona will emerge.

**Fig. 2:**
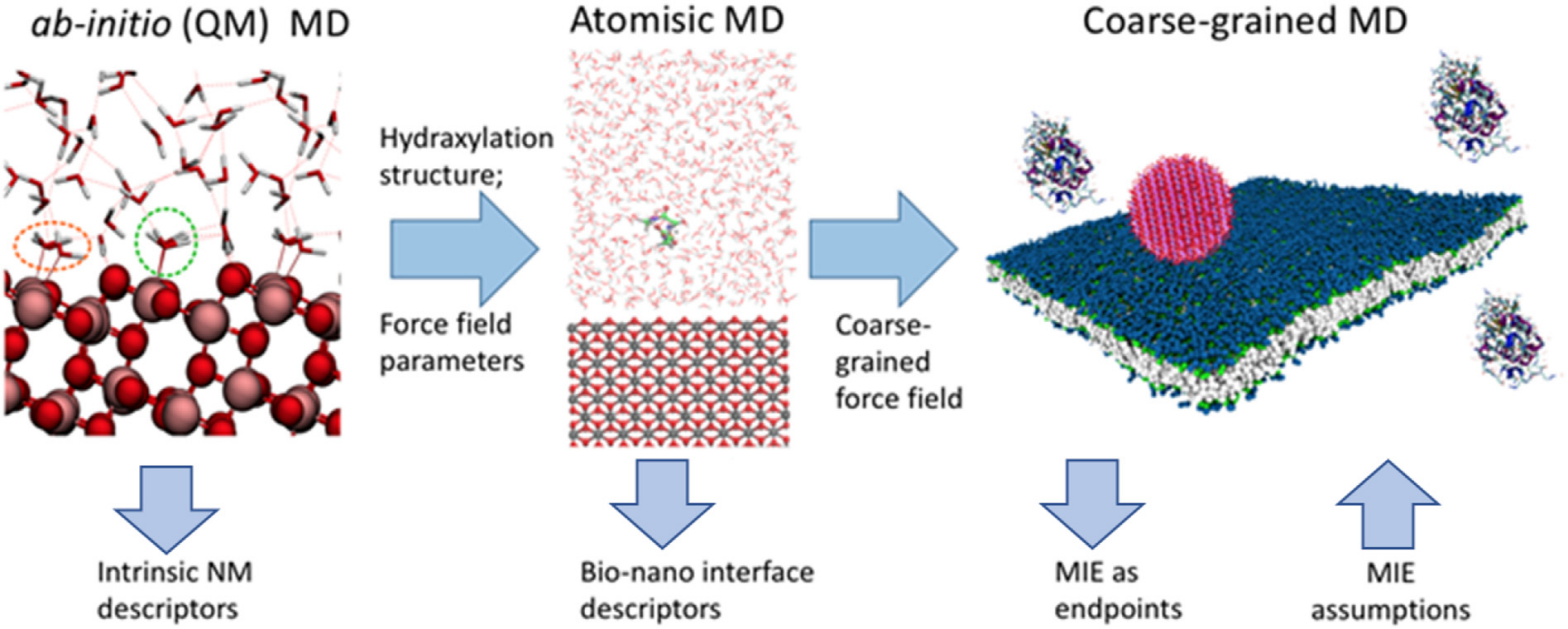
Schematic illustration of the NanoSolveIT approach to multi-scale modelling of NM interactions with biomolecules to form the biomolecule corona which provides the biological identify to the NM and determines its subsequent uptake and impacts in cells and organisms.

NanoSolveIT recently proposed a multiscale modelling scheme that enables modelling of large molecular assemblies in both length and time domains, which is not achievable by traditional atomistic simulations. This allows information on NM and biomolecule specificity to be preserved [82], [81], [129] whilst also enabling calculations in reasonable times. The NanoSolveIT method (shown schematically in Fig. 2) uses a systematic CG method that includes:

• parameterization of the atomistic force-field for the NM;
• calculation of interactions of the biomolecule building blocks (amino acids, lipid segments, DNA bases) with the surface of the NM and interaction between the building blocks at the atomistic level under specified conditions;
• parameterization of the CG force field for biomolecule building blocks and construction of a NM of arbitrary size and shape;
• CG modelling of interaction of entire biomolecules with the NMs’ surface and calculation of preferred orientation and the mean adsorption energies for the bound biomolecules;
• further coarse graining for lipids and proteins to make united amino acid blocks and study competitive adsorption and bionano-interface structure.

The multiscale modelling framework proposed by NanoSolveIT enables calculation of descriptors and properties of the bionano interface for a large number of biomolecules in a short time. The new properties include those for proteins (principal moments of inertia, charge, dipole moment, hydrophobicity indices, and the solvent accessible area) and those for interaction with NM (Hamaker constants for residues, their mean adsorption energies, and the overall adsorption energy for the protein globule or lipid molecule) [82]. These properties will be used to produce interaction fingerprints for arbitrary NMs with respect to specified biological activities and will thus provide key information for toxicologically relevant predictive modelling, e.g., for predicting NM ability to induce an AOP via MIE or KE.

Several models describing how binding to NM surfaces affects protein conformations and subsequent recognition behaviour have appeared in the literature. These will be very useful for connecting corona composition to molecular initiating and other key events in AOPs. Multiscale MD simulations of a single NM with the protein ubiquitin demonstrated that ubiquitin competed with citrates for the NN surface. At a high protein/NM stoichiometry, ubiquitins formed a multi-layer corona on the particle surface, with the proteins exhibiting conformational changes that included destabilization of α-helices and increased β-sheet content of the proteins [22]. A significant challenge with this approach is that it is unknown whether this protein binds under competitive conditions (e.g. in plasma), and whether these conformational changes occur in real systems. However, the correlation between unfolding of a specific protein and receptor activation has also been demonstrated experimentally and modelled using MD. Ding et al. showed that specific sizes of negatively charged poly(acrylic acid)-conjugated gold NPs bound to, and induced unfolding of, fibrinogen (Fg). This promoted interaction with the integrin receptor, Mac-1, leading to increased NF-κB signalling and release of inflammatory cytokines [21]. Building on this work, Kharazian et al. used MD simulation to investigate how poly(acrylic acid) coats a gold NM surface. The root-mean-square deviation (RMSD), radius of gyration (Rg) and solvent accessible surface area (SASA) properties from the calculations showed that the gold surface can induce Fg conformational changes favouring an inflammation response [62]. They suggest that the integrity of coatings on ultra-small gold NMs are compromised by the large surface curvature, and that surface coatings may be degraded by physiological activity. Other modelling studies have also assessed biomolecule conformation in NM coronas and, where relevant, these approaches will be integrated into the NanoSolveIT toolbox.

#### 2.3.3 Connecting NM-coronas to biological impacts/AOPs

Data exchange and reuse puts significant limits on how we represent the biology and chemistry of NMs. For example, to compare the risk of two NMs, it is necessary to know whether they are chemically similar and if they are behaving biologically in the same way (e.g. via read across studies). To ensure data reuse is possible, the NM data representations must be interoperable, i.e. both humans and machines must be able to make such chemical and biological comparisons. The bioinformatics and cheminformatics communities have developed extensive methods to perform such tasks. To be reusable, data must meet community standards around data quality [92], be interoperable (i.e. be able to interact with other databases), be machine accessible (i.e. be annotated to allow computers to understand the individual datapoints), not be hidden behind firewalls and be findable by search engines and models. Recently, these ideas were summarized in the FAIR principles [181] and the need for interoperability and data linking was recently outlined in a position paper [60]. A key step for FAIRness was the development of a common NMs ontology to facilitate data interoperability [48], [100].

Several recent studies focused on developing models of NM-related properties and biological effects [10], [123], [142], [159], [160], [161], [163]. For example, a set of 18 NMs were studied by Wang et al. [178] and showed that factors such as metal content, surface charge, and particle morphology induce high toxicity. Melagraki et al. developed, a predictive classification model based on OECD principles, for the toxicological assessment of iron oxide NMs with different cores, coatings and surface modifications based on a number of different properties including size, relaxivities, zeta potential and type of coating [97]. More recently a predictive nanoinformatics model, validated according to the OECD principles, has been developed for the prediction of the protein binding and the cytotoxicity of functionalized multi-walled carbon nanotubes [172].

Numerous other as yet-unexplored features may also be important for generating adverse effects from NMs. For instance, there is evidence to suggest that NMs, especially in the lower nm range, penetrate biological membranes and are able to reach organs that are otherwise inaccessible for larger substances [14]. Exposure to specific NMs has also been demonstrated to cause adverse biological effects by increased production of ROS, such as oxyradicals [79], [103], [112]. However, some NMs may be less harmful than their corresponding bulk forms in some instances [61] and even two NMs from the same source, with similar sizes and chemical compositions may exhibit diverse effects [121]. Clearly, factors other than size, shape and surface area are also important in controlling the interactions and effects of NMs in biological systems. For example, Zhang et al. proposed that the higher toxicity of fumed silica relative to Stöber silica stems from the formers’ intrinsic population of strained three-membered rings along with its chainlike aggregation and hydroxyl content [189].

Surface coatings are crucial for control of both useful and adverse effects of NMs. They provide a great opportunity to improve materials through rational design, by enhancing a useful property and reducing adverse effects. This approach has been demonstrated for multi-walled carbon nanotubes (MWCNT) and asbestos fibers, where structural similarities suggested potentially harmful effects according to the so-called ‘fiber paradigm’. The similarities in the two structures guided a pilot study, which showed that when long MWCNTs are injected into the abdominal cavity of mice, asbestos-like pathogenic effects can be induced [125]. However, less rigid forms of CNTs had lower toxicity [105], suggesting rigidity as a potential design parameter. A larger pulmonary toxicological study of 10 MWCNT demonstrated increased inflammation and lower genotoxicity with increased surface area (decreasing CNT diameter) and some reduction in inflammation with –OH and –COOH surface functionalization [126]. Toxicogenomic markers of MWCNT-induced fibrosis [128] and acute phase response as a predictor of cardiovascular disease [127] have also been identified. Thus, future experimental and modelling studies will explore the correlations between a wider range of NMs parameters, functionalizations, corona compositions, cellular associations and AOPs.

### 2.4 Predictive nanoinformatics modelling (using artificial intelligence methodologies)

Increasing use of NMs has escalated concern about their potential risk to the environment and human health. The RA of NMs has traditionally been based on a variety of *in vitro* and *in vivo* assays, which are typically expensive, labour-intensive and time-consuming. *In vivo* animal testing has been constrained by ethical considerations to follow the 3Rs rule aiming to replace, reduce, and refine the use of animals for scientific purposes [140], and from 2013, the use of animals is banned for safety assessment of cosmetic products [28]. Due to the multitude and variety of NMs increasingly exploited in our daily life, it is impossible to assess *in vitro* or *in vivo* potential risk of all NMs on a case-by-case basis [33]. In addition, application of the full REACH information requirements for every single variant of a given NM regarding particle size, shape, or surface properties, which accelerate the toxic nature, would lead to an insurmountable amount of testing [30].

To complement existing toxicity tests, computational approaches (*in silico* models) have been proposed as useful alternatives to predict *in silico* the potential hazard of NMs, to reduce time and cost of nanosafety assessments and provide input for design of safe and functional NMs. QSARs, as *in silico* models, have been developed to predict biological activities, including the toxicity of various substances [134], mainly based on molecular structure [96], SMILES [179], and international chemical identifiers (InChI) [49]. However, such classic QSAR approaches have shown many limitations in predicting the toxicity of NMs due to the lack of standardized databases on their structures together with PChem parameters/descriptors and various toxicity endpoints [119]. In addition, as the toxic effects of NMs vary according to their PChem properties and the same type of NMs exhibit diverse toxic effects under different biochemical conditions (e.g., cell line, cell species, etc.) [39], [124], [191], making classic QSAR modelling difficult.

To overcome these challenges and establish relationships between nano-descriptors, which express the novel and size-dependent properties of NMs, and toxicological adverse effects triggered by NMs, various modelling approaches based on statistics and machine learning (ML) have been proposed to predict qualitatively or quantitatively *in silico* endpoints. Generally, developing predictive models includes the following major steps [114]:

(1) data collection that contains associations between NMs and endpoints;
(2) data preprocessing to transform the raw data into a useful and efficient format and to improve the quality of the raw data and reduce batch effects between experiments;
(3) selection of nano-descriptors (fingerprint) predictively linked to functionality and hazard of NMs;
(4) predictive model development and validation;
(5) mechanism interpretation;
(6) and definition of the applicability domain of the model.

Early ML models for predicting NMs properties, such as protein corona (PC), have begun to appear in the literature For example, a random forest classification approach has been shown to model PC populations for an array of NMs properties and reaction conditions, while providing insight into feature importance to define which aspects of protein, NM, and solvent chemistry are the most important to defining the PC population [31]. The model has the potential for prediction of NM PC fingerprints across a wide range of NMs, protein populations, and reaction conditions. Varsou et al. presented a novel read-across ML methodology based on a mathematical optimization approach for the prediction of NMs toxic effects [170]. This is an area of intensive research currently and numerous advances are expected in the near future.

#### 2.4.1 Calculated toxicity predictors for ML models

Effective prediction of NMs health and environmental risks requires utilization of existing or newly generated data to develop *in silico* hazard assessment models based on refined hazard-correlated endpoints. Achieving this requires establishment of a unified methodology for predicting the risks related to use of NMs, building on a sustainable multi-scale nanoinformatics framework, which links existing and emerging data and integrates, facilitates and advances the current state-of-the-art *in silico* modelling and predictive toxicology approaches.

A major objective of nanoinformatics is thus the development of *in silico* methods for predicting biological activities of NMs. These models will be trained on NM structural information (encoded by nanostructure descriptors) and PChem features (properties). Therefore, one of the most important and difficult challenges is to devise nano-specific descriptors that encode these features. When dealing with large numbers of NMs, it is important to use calculated descriptors, as those that require experimental measurement will become less useful and more expensive to generate as the data set sizes grow. Initial strategies for nano-specific descriptors include those derived from SMILES strings, the periodic table, simplex representations of molecular structure, liquid drop model descriptors and those derived from NanoJava applets [27], [58], [69], [150], [155]). Additional potentially valuable information about morphological features of NMs will be extracted from microscopy images, e.g. size, area, circularity, and aspect ratio, surface topography [113] and the use of modern automatic image analysis software tools such as NanoImage and NanoXtract developed specifically for NMs. For instance, utilizing NanoXtract [171], a NM Image Analysis tool powered by the Enalos Cloud Platform, users can analyze TEM microscopy images and extract useful descriptors that can be used in a future step as inputs to predictive models. Within a simple and user-friendly interface, the user can upload a single TEM image of a specific NM and with just a few clicks to obtain a set of NM image descriptors displayed on the screen or downloaded as a .csv file. These image descriptors can be explored by developing nanoQSAR models, either within the Enalos Cloud platform or using in-house models, to identify those descriptors most predictive of NM behaviour and /or biological effects.

#### 2.4.2 Meta models to integrate across scales

An optimum set of nano-specific descriptors will enable generation of models describing the relationships between them and experimental and computed NM properties (intrinsic and extrinsic) and a wide variety of biological endpoints. In classic nano-QSAR modelling, both structural information and properties are treated as independent variables. However, NanoSolveIT will also use meta-models, where a property (e.g. hydrophobicity, NM-biomolecule interactions, surface activity, adsorption properties) is first derived from descriptors, e.g.:

$$\text{property}=f\left(\text{descriptors}\;\text{of}\;\text{NMs}\right).$$

**Fig. 3:**
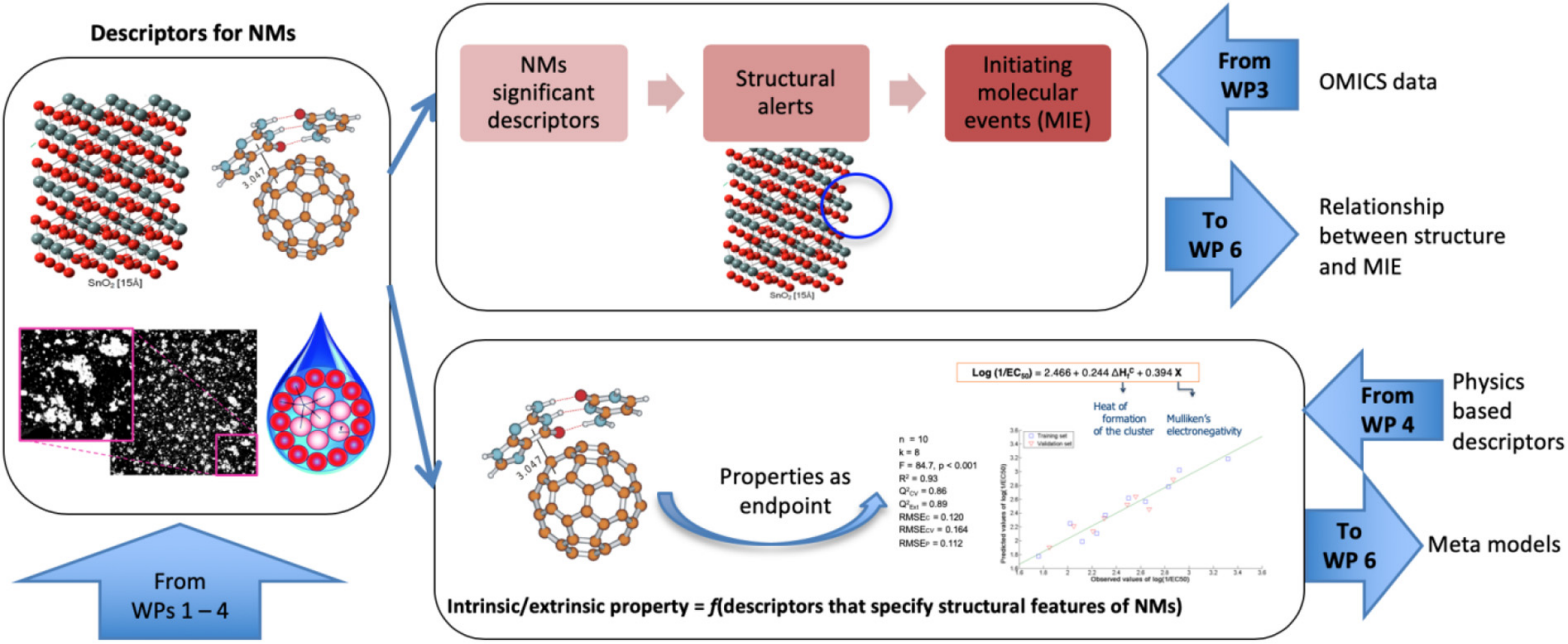
NanoSolveIT meta models.

This meta model ML approach will also allow rapid estimation of properties of many NMs that would normally be obtained from computationally demanding QC calculations and/or molecular dynamics simulations. Furthermore, meta models will be used to provide deeper insight into the toxic effect induced by NMs, from the mapping between NM properties (obtained from simulation), uptake, internalization and Physiologically Based Pharmacokinetics (PBPK) considerations, and the observed toxic effects, as shown in Fig. 3. This will be achieved by analyzing which structural, PChem, or meta model-derived property has the largest impact on adverse biological outcomes induced by NMs. This approach also allows implementation of the safe-by-design concept, where ML models of commercially valuable properties and adverse biological effects are used to rationally design NMs with optimum performance and greatest safety. These methods are also valuable for research groups and institutions that focus more on NM synthesis rather than RA.

Thus, NanoSolveIT proposes a new, broader view of nanoinformatics. All ML models developed in the project will be trained on heterogeneous data from multiple sources (experimental characterization, image analysis, computational simulation, omics data) that have, until now, been treated individually. These integrated NM “fingerprints” will contain maximal information required to build statistically robust and optimally predictive models of biological activity. Optimal predictivity will be ensured by the use of sparse feature selection (MLREM, LASSO), Bayesian regularization to control model complexity (LTU, Biomodeller), and automated processes included in the RRegrs R package [169]. The RRegrs package automatically selects the next algorithm for each case from 10 different statistical and ML methodologies (e.g. multiple regression, lasso, support vector machines, and artificial neural networks). Computational efficiency will be achieved by the use of Graphical Processing Unit (GPU) algorithms through the Enalos + toolkit [173]. This improves big data handling and accelerates computation. All models will be built according to OECD guidelines, where feasible. All models will be validated using test sets, cross-validation, bootstrapping and Y-scrambling as appropriate [3]. Domains of applicability for models will be calculated by established methods, such as the leverage method and the Euclidean distance to the training set.

### 2.5 NM human and environmental RA

The ultimate goal of multi-scale nanoinformatics modelling is the generation of a computational platform that integrates *in silico* approaches to NMs safety testing and assessment into an overall risk framework or IATA. The IATA will provide a systematic framework for the integration of the data and models used and/or produced during the project’s lifetime. The IATA will be configured to ensure correct mapping of the sequence with which the developed and integrated tools are run thereby ensuring that the inputs and outputs from the various tools are harmonized and interoperable. This will ensure proper tool function and optimization of the initial data input based on the desired outcomes in order to answer specific stakeholder needs (safe-by-design, exposure, hazard and RA) and make predictions regarding the safety, mode of action, and likelihood of a NM triggering a specific AOP. NanoSolveIT is building on previous efforts to map and evaluate a range of NMs-specific risk assessment tools or so-called “new approach methodologies” in terms of their ability to provide “added value” and “decision support” as well as their status in terms of whether they are ready for implementation or require further exploration and development [109].

One of the key functions of the IATA will be the development of NM fingerprints - predictive and informative PChem, biological and computational descriptors that describe a set functionality, i.e. the minimum set of descriptors required as input to predict specific NM functionalities. The IATA will focus on those parameters that are easy to measure/calculate on a regular basis during every day experimental and computational practice. Classification approaches of relevant descriptors as intrinsic (do not change irrespective of the exposure route, release pathway) or extrinsic (context-dependent) and the stability of potential transformations will be also incorporated into the IATA [89]. This will reduce the experimental/computational workload to derive specific descriptors and subsequently the amount of information needed to create robust predictions, especially in terms of safe-by-design. In the case where a NM is classified as hazardous, the generation of additional parameters will be proposed to refine the ultimate hazard and RA. As a result, a certain set of exposure and toxicokinetics models will be integrated and linked within the IATA. These models will act in a synergistic way to fulfil the requirements of the NanoSolveIT framework.

In every case, NanoSolveIT will look to deliver informative and validated predictive models with clear definitions of their domains of applicability. For the NanoSolveIT IATA to be successful, these models need to act synergistically. This will require standardized modelling approaches and workflows that take into account the OECD validation principles [114] and recommendations from the European Materials Modelling Council [26].

To achieve the required synergism, a clearly defined workflow has been established, which includes the following steps:

1. Selection of modelling tools for the NanoSolveIT IATA, appropriate to the needs of stakeholders.
2. Evaluation of model parameterization needs to identify the required parameters for each tool to support the NM fingerprint and the IATA.
3. Generation of plausible and useful exposure and environmental release scenarios covering the entire lifecycle assessment of NMs. These will be based on data from recent, ongoing and future studies covering NMs’ release points during production, use and disposal.
4. Technical solutions for incorporation of the various modelling tools into the NanoSolveIT IATA and e-Platform.

To achieve these ambitious goals and demonstrate the utility of the IATA to the research, industrial and regulatory communities, NanoSolveIT is building on recent advances from e-infrastructure projects OpenRiskNet and NanoCommons. For example, a key challenge is integration and alignment of different sources of information to allow use of weight of evidence approaches and evaluation of uncertainties in the information. Here approaches already implemented in NanoCommons will be leveraged. For example, NanoCommons has already integrated the similarity scoring and data quality modules of the GUIDEnano platform for RA and risk management of NMs, which are essential for weight of evidence analysis. Similarly, NanoCommons has supported the OECD WPMN project on NanoAOPs [117], including updating and expanding the database for the KEs identified for the tissue injury adverse outcome. It is building on that experience to develop KE-specific data capture templates for electronic notebooks, harmonizing data capture and data curation approaches, and text mining tools to speed up compilation of literature datasets. Integration of tools for searching the NanoWiki and identifying KEs is also currently underway in NanoCommons, and will be incorporated into NanoSolveIT. NanoCommons has already demonstrated approaches for integration of datasets, their curation and quality assurance, using its two main modelling platforms, namely Enalos tools via KNIME and Jaqpot via Jupyter notebooks. Currently these NanoCommons deliverables are under review by the European Commission but will be made publicly available via the NanoCommons website as soon as possible, to allow wider community adoption of the approaches, as per all the public deliverables. Datasets can be pulled from a range of existing databases such as PubChem, UniChem, UniProt etc. via dedicated Enalos APIs (through KNIME nodes) and Python and R-scripts for Jaqpot.

A final demonstration of the utility of the NanoSolveIT IATA will be via case studies that showcase the various components and the overall approach. These will include existing OECD IATA case studies and assessment of their applicability to NMs, as well as a case study on developing NanoAOPs for genotoxicity and potentially for accelerated ageing in *Daphnia magna* based on the existing extensive datasets available within the NanoSolveIT consortium.

The three OECD IATA case studies identified for NanoSolveIT are: –

1. Prioritization of chemicals using IATA-based Ecological Risk Classification (Prioritization of chemicals / Ecotoxicity) – NanoSolveIT will adapt for NMs;

2. Case study on grouping & read-across for NMs genotoxicity of nano-TiO_2_ (Grouping / Genotoxicity) – NanoSolveIT will assess the ability of our models / IATA to reproduce (improve on) the findings;

3. A Case Study on Use of IATA for Sub-Chronic Repeated-Dose Toxicity of Simple Aryl Alcohol Alkyl Carboxylic Esters: Read-Across (Grouping (Read-across) / Repeated dose toxicity) – NanoSolveIT will adapt this for NMs.

It is also worth noting that expert judgement is an essential part of the IATA process. Both main computational platforms being used to underpin the NanoSolveIT IATA, namely Enalos tools via KNIME and Jaqpot via Jupyter notebooks, are fully configurable as nodes. This allows the establishment of pre-configured workflows that need minimal input from the user, but that allow expert intervention and decisions where required. For example, workflows can include opportunities to download the raw and transformed datasets for quality assurance purposes, analysis of data quality reports, and other checks prior to proceeding to the next step. Additionally, Bayesian belief network are being integrated into the IATA to automatically manage expert judgement and modelling reliability. This allows analysis of the accuracy of the expertise and ensures that the expert opinion does not bias model expectations [19], [148]. Bayesian networks are excellent quantitative and tractable RA tools that can handle and augment datasets with missing values by incorporating expert knowledge and judgment. They have been used successfully for developing IATAs for simple chemicals, and recently applications to NM RA have been reported [104].

### 2.6 NM cloud platform

**Fig. 4:**
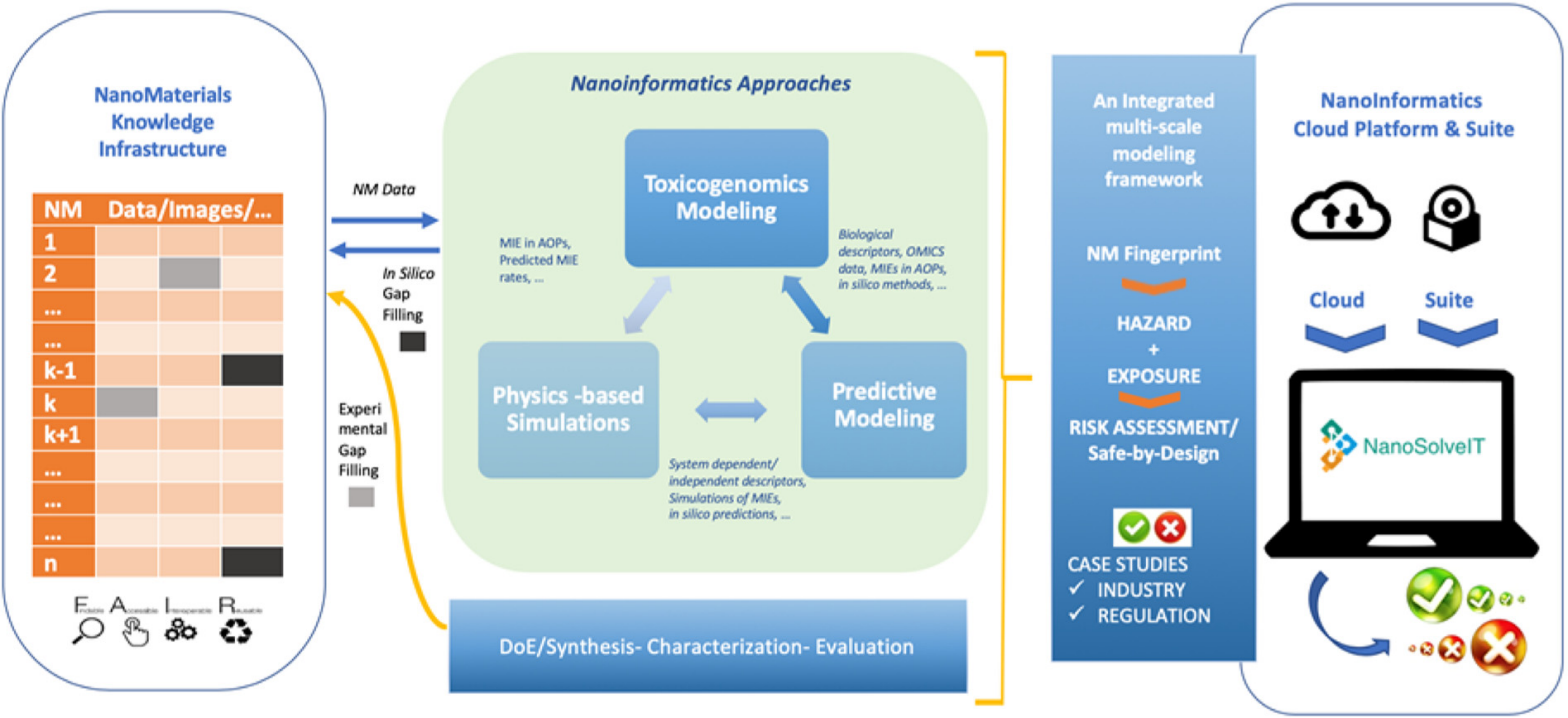
NanoSolveIT platform integrating all of the various omics, materials and machine learning and meta models into an harmonized platform for NMs properties, exposure, hazard and risk prediction, safe-by-design and *in silico* NMs toxicology. All available data from the contributing projects and literature, including acute and chronic toxicity data, and a strong focus on regulatory-relevant endpoints, will be incorporated into the database.

A major outcome of the NanoSolveIT project is the e-platform that will be made available to the community as a cloud application accessible via the web or which can be installed as a standalone platform on local servers of interested stakeholders (see Fig. 4). This platform provides all the computational modelling tools and functionalities developed during the project under a common framework and with an optimized workflow for input of data and generation of predictions in response to specific stakeholder queries. These individual models and tools will also be available as separate components for specific applications and needs, but the ultimate goal is to integrate the tools as much as possible, to allow the implementation of complete computational IATA workflows.

The system architecture of the e-platform will be efficient, user-friendly, extensible, easy to maintain and open to the community for integration with other tools and databases. Our vision is to develop a central and easily extensible web-based platform of secured sustainability that will be customizable to specific user requirements and will address both current and future research and regulatory questions and needs.

To achieve all these objectives, state-of-the art tools and best practices in developing web applications and platforms will be leveraged. An open microservice architecture relying on the OpenAPI specifications for constructing and testing the APIs, and the REST (Representational State Transfer) architectural style for designing distributed systems will be utilized. This will consider relevant operational aspects of the platform such as extensibility, scalability, and elasticity.

The development of the NanoSolveIT e-platform will build on, incorporate and extend two of the most comprehensive platforms available in the nanosafety community, namely Jaqpot [18] and Enalos (NovaMechanics [108], [171]). These are already used for developing, hosting, sharing and exchanging predictive models. Additionally, it will leverage exposure and fate models developed in recent H2020 projects, such as the NANOFASE model for environmental fate and effects, a multi-box aerosol-dynamic model [57] that with further development and testing will be used to calculate the size-resolved NM inhalation exposure and dose, and the GUIDEnano tool for exposure, hazard and RA which has already been integrated into the NanoCommons research infrastructure.

For simulation of the biokinetics of NMs, i.e. the administration, distribution, metabolism and excretion of NMs when they enter an organism, the NanoSolveIT platform will integrate and extend functionalities that have been developed in the Jaqpot modelling environment. Detailed PBPK / Toxicokinetics models will be provided as web applications, ready to simulate various NM external exposure scenarios (e.g. single dose, multiple doses, repeated dose etc.) and the physiological parameters of the individual or the group of people who are exposed. The concentration–time responses in the various organs and tissues of the organism will be automatically generated as interpretable and visual tables and graphs. By connecting the exposure and the biokinetics models, NanoSolveIT will develop integrated workflows that model how an external exposure scenario affects the internal exposure, i.e. the NM concentration in specific tissues of interest. By further integrating and interlinking these tools with models that can predict hazard at the AOP level, the NanoSolveIT platform will be able to predict whether an exposure scenario can initiate a specific AOP in a specific individual.

Hazard assessment services will be part of the NanoSolveIT platform, by providing ready-to-use web implementations of nano QSAR models and the read-across approaches that are being developed in the project. By integrating ML libraries, practically all state-of-the-art statistical and ML methodologies will be available for model building. Custom-made solutions, such as implementation of read-across methods or specific ML algorithms will also be possible if they are compatible with the architecture and the design principles of the platform. Hazards and RA tools developed in previous projects (e.g., NanoCommons & NanoMILE tools hosted on the Enalos Cloud Platform) are already available as user-friendly applications and will be further expanded. Several of the above mentioned predictive nanoinformatics models [1], [97], [98], [172] are already hosted in the Enalos Cloud Platform and are publicly available. The Enalos Cloud Platform [174], is based on web semantic cheminformatics and nanoinformatics application, with the ability to host any predictive model as a web service with a user friendly user interface (NovaMechanics [108]). Web applications are invaluable for computer-aided design of NMs since they can be used to predict the activity of new NMs prior to synthesis and the biological evaluation. Furthermore, ‘safe-by-design’ paradigms can be easily constructed for a systematic study of the effects of NM structural modifications and properties on several relevant biological responses and crucial properties.

Deep Neural Networks and deep learning algorithms have received much attention in the ML community and increasingly many other scientific, technological, business, and medical spheres over the last few years. These methods are closely related to or incorporated into big data analysis, especially for large volumes of high-resolution images. Applications of deep learning in the nanosafety discipline are so far quite rare. The NanoSolveIT project will fill this technology gap by developing infrastructure for implementation of deep learning models as web services. Specific demonstrator applications will be provided to showcase the capabilities of these technologies, for example use of only image data to predict whether an organism is affected or damaged by the presence of an NM. These AI capabilities of the platform will be supported by graphics processing units (GPUs).

The NanoSolveIT e-platform will be seamlessly integrated with the project knowledge base (Section 2.1.3) that will provide the necessary information for developing, testing and finally using the models for predictive purposes. All modelling components and the complete platform will comply with the ontological annotations used throughout the project and particularly in the development of the knowledge base. Model predictions will be used to fill gaps in the NanoSolveIT knowledge base, due to lack of experimental data. Thus, the system is designed in a manner that allows continuous improvement as new data emerges, and integrates seamlessly experimental and computational data, thereby constantly enriching the underpinning knowledge base.

### 2.7 Knowledge transfer and communication with stakeholders and RA bodies

Due to the current lack of *in vitro* and *in vivo* data risk assessors seek reliable *in silico* approaches, derived from validated or scientifically-valid *in silico* models, grouping and read-across, PBPK and toxicokinetic modelling [7], and for support scientifically based regulatory nano-risk governance. The current state-of-the-art on alternative testing strategies in RA of engineered NMs was published by the OECD [116]. A tiered approach based on non-testing and *in vitro* methods has been suggested for the prediction of realistic biological outcomes when used in a weight of evidence (WoE) approach. Several frameworks and *in silico* approaches been proposed but even so risk assessors still consider *in silico* modelling tools to be at an elementary stage for NMs [7], [25], [116] stressing the need to develop and provide a set of standard predictive models with defined parameters that can accurately and efficiently predict human and ecological toxicity of NM with minimal biological experimentation.

The NanoSolveIT advanced nanoinformatics approach addresses these needs by developing integrated models within a benchmarked IATA for nanosafety assessment. The innovative modelling techniques and tools will be integrated within NanoSolveIT IATA and then incorporated into a sustainable interoperable product, the NanoSolveIT e-platform. NanoSolveIT will deliver an innovative alternative testing strategy that is less reliant on animal testing for NMs RA using predominantly *in silico* derived NM descriptors to generate validated predictive models for NMs properties and adverse effects.

To accelerate implementation of the advances in nanoinformatics, engagement with regulators and policy makers is needed, to create dialog and transfer NanoSolveIT knowledge. Within NanoSolveIT a strong communication, dissemination and exploitation strategy for spreading and circulating information, managing effective communication and delivery of information, and up-skilling of relevant stakeholders enabling them to make use of and benefit from the created knowledge and resources, is implemented in parallel with the technological advances. This will ensure long-term exploitation of the NanoSolveIT IATA and outcomes, across the full range of regulatory bodies (e.g., ECHA, EFSA, SCCS, OECD, etc.). By direct communication with regulatory bodies, including risk assessors as members of the Advisory board, and using advanced communication tools to engage all relevant stakeholders NanoSolveIT aims to create common ground for exchange of information between scientists, industry, risk assessors, regulators, and policy makers. For example, several stakeholder workshops have been planned, aligning with similar nanoinformatics activities and parallel risk governance projects, to ensure that tools developed within NanoSolveIT will become utilized as an essential element for supporting industrial and regulatory nano-risk governance.

NanoSolveIT also offers the opportunity for alignment and harmonization of international activities into the ongoing and emerging activities in Europe. This will be achieved through the participation of international partners who will participate in the benchmarking of the various tools and models and the assessment of their suitability for addressing different safe-by-design, grouping and categorization, and predictive (eco)toxicity questions via the Round Robin testing. This activity also relies on meaningful engagement with international activities such as the OECD, including via the Malta Initiative, on the revision of test guidelines and development of predictive modelling approaches.

## 3 Summary and outlook

Significant advances have been made during the last decade in the field of nanoinformatics, which have resulted in the development of various modelling frameworks, data platforms and knowledge infrastructures, and *in silico* tools for generating meaningful hazard and RA predictions for NMs. A short analysis of the recent advances in the distinctive nanoinformatics fields has been provided here, identifying the state-of-the-art, the current gaps in research and potential challenges to be solved by the development of innovative techniques and methods.

First, the issues of data quality, reliability and accessibility have to be addressed in a harmonized manner by ensuring that all NMs data platforms are connected and follow the same standards in order to allow easy access to curated, relevant data, under the FAIR principles. Quality and reliability assessment of data for NMs remains an important challenge and should be performed as early as possible in the data generation process, preferably at the point of data generation as promoted by the NanoCommons nanosafety research infrastructure [120].

Secondly, scientific tools for nanoinformatics have been, and continue to be, developed. The effort to collect and link the available tools under state-of-the-art platforms is the next step, closely related to the efforts to perform holistic risk governance processes for NMs. This would increase the accessibility of the tools and would allow possible validation of the available tools via benchmarking and comparison of predictions. This would provide high interoperability and facilitate reuse of data.

Addressing these challenges, NanoSolveIT is developing a broad spectrum of diverse but interlinked advanced physics–based, omics-based and data-driven (AI, deep learning) models that work synergistically exchanging inputs and outputs and which together constitute a multi-scale *in silico* IATA for evaluation of the environmental health and safety of NMs. This will be achieved through the development and use of an extraordinary breadth of integrated models ranging from NM-biomolecule and cell interactions, models for ‘omics analysis and AOP generation, release and exposure models, toxicokinetics and PBPK models, and ecotoxicity prediction, all delivered within a benchmarked IATA for nanosafety assessment, to enable safe-by-design NM development and NMs Risk Governance. The NanoSolveIT IATA will be implemented as a decision support system packaged both as a standalone software and a Cloud platform.

Key impacts of the NanoSolveIT models and *in silico* IATA will be a direct reduction in the need for animal testing and a concurrent increase in regulatory, industry and consumer confidence in the predictive capacity of the nanoinformatics nanosafety models. The *in silico* IATA will support the implementation of “safe-built-by-design” approaches in industry by applying cost-effective testing platforms for exposure, hazard and RA.

Acknowledging the complexity of the challenge, complete achievement of this vision is likely to require 10 years of research and investment, however, NanoSolveIT anticipates having a first version of the concept implemented by early 2023, utilizing open access approaches where possible to allow further community development and continuous enrichment of the knowledgebase underpinning the models. Exciting times are ahead for the nanosafety Nanoinformatics community.

## CRediT authorship contribution statement

**Antreas Afantitis:** Conceptualization, Supervision, Writing - original draft, Writing - review & editing. **Georgia Melagraki:** Conceptualization, Supervision, Writing - original draft, Writing - review & editing. **Panagiotis Isigonis:** Writing - original draft. **Andreas Tsoumanis:** Writing - original draft. **Dimitra Danai Varsou:** Writing - original draft. **Eugenia Valsami-Jones:** Writing - original draft, Writing - review & editing. **Anastasios Papadiamantis:** Writing - original draft. **Laura-Jayne A. Ellis:** Writing - original draft. **Haralambos Sarimveis:** Conceptualization, Supervision, Writing - original draft, Writing - review & editing. **Philip Doganis:** Conceptualization, Writing - original draft, Writing - review & editing. **Pantelis Karatzas:** Writing - original draft. **Periklis Tsiros:** Writing - original draft. **Irene Liampa:** Writing - original draft. **Vladimir Lobaskin:** Conceptualization, Supervision, Writing - original draft, Writing - review & editing. **Dario Greco:** Conceptualization, Supervision, Writing - original draft, Writing - review & editing. **Angela Serra:** Writing - original draft. **Pia Anneli Sofia Kinaret:** Writing - original draft. **Laura Aliisa Saarimäki:** Writing - original draft. **Roland Grafström:** Conceptualization, Supervision, Writing - review & editing. **Pekka Kohonen:** Writing - original draft. **Penny Nymark:** Writing - original draft, Writing - review & editing. **Egon Willighagen:** Conceptualization, Supervision, Writing - review & editing. **Tomasz Puzyn:** Conceptualization, Supervision, Writing - review & editing. **Anna Rybinska-Fryca:** Writing - original draft. **Alexander Lyubartsev:** Writing - original draft. **Keld Alstrup Jensen:** Writing - original draft. **Jan Gerit Brandenburg:** Writing - review & editing. **Stephen Lofts:** Conceptualization, Writing - review & editing. **Claus Svendsen:** Conceptualization, Supervision. **Samuel Harrison:** Writing - original draft. **Dieter Maier:** Conceptualization, Writing - original draft, Writing - review & editing. **Kaido Tamm:** Conceptualization, Writing - original draft, Writing - review & editing. **Jaak Jänes:** Writing - review & editing. **Lauri Sikk:** Writing - review & editing. **Maria Dusinska:** Conceptualization, Writing - original draft, Writing - review & editing. **Eleonora Longhin:** Writing - original draft. **Elise Rundén-Pran:** Writing - original draft. **Espen Mariussen:** Writing - original draft. **Naouale El Yamani:** Writing - review & editing. **Wolfgang Unger:** Writing - original draft. **Jörg Radnik:** Writing - original draft, Writing - review & editing. **Alexander Tropsha:** Writing - original draft, Writing - review & editing. **Yoram Cohen:** Writing - original draft, Writing - review & editing. **Jerzy Leszczynski:** Writing - original draft, Writing - review & editing. **Christine Ogilvie Hendren:** Writing - original draft, Writing - review & editing. **Mark Wiesner:** Writing - original draft, Writing - review & editing. **David Winkler:** Conceptualization, Writing - original draft, Writing - review & editing. **Noriyuki Suzuki:** Writing - original draft, Writing - review & editing. **Tae Hyun Yoon:** Conceptualization, Writing - original draft, Writing - review & editing. **Jang-Sik Choi:** Writing - original draft, Writing - review & editing. **Natasha Sanabria:** Writing - original draft, Writing - review & editing. **Mary Gulumian:** Conceptualization, Writing - original draft, Writing - review & editing. **Iseult Lynch:** Conceptualization, Supervision, Writing - original draft, Writing - review & editing.
